# Patients’ Experiences of Clozapine for Treatment-Resistant Schizophrenia: A Systematic Review

**DOI:** 10.1093/schizbullopen/sgac042

**Published:** 2022-07-10

**Authors:** Steven Parkes, Bethany Mantell, Ebenezer Oloyede, Graham Blackman

**Affiliations:** Department of Psychosis Studies, Institute of Psychiatry, Psychology and Neuroscience, King’s College London, London, UK; Department of Psychosis Studies, Institute of Psychiatry, Psychology and Neuroscience, King’s College London, London, UK; South London and Maudsley NHS Foundation Trust, London, UK; Department of Psychosis Studies, Institute of Psychiatry, Psychology and Neuroscience, King’s College London, London, UK; South London and Maudsley NHS Foundation Trust, London, UK; Department of Psychosis Studies, Institute of Psychiatry, Psychology and Neuroscience, King’s College London, London, UK; South London and Maudsley NHS Foundation Trust, London, UK

**Keywords:** psychosis, antipsychotics, treatment-resistance, subjective experience

## Abstract

**Background:**

Clozapine is the most effective antipsychotic for patients with treatment-resistant schizophrenia (TRS), however, it remains widely under-utilized in clinical practice. To date, relatively little attention has been given to patients’ experience of clozapine. By synthesizing the existing literature, we sought to determine the experiences of patients with TRS treated with clozapine.

**Methods:**

A systematic review was conducted on Embase, Medline, PsychInfo, and PubMed databases for studies from 1956 to 2021. English language studies and those based on adult patients prescribed clozapine for TRS were included.

**Results:**

Thirteen studies were included with a total of 1487 patients and a narrative synthesis was performed. Overall, most patients reported positive experiences of clozapine, with generally high levels of satisfaction, alongside symptom improvement and preference over previous medications. Negative experiences of clozapine were less common, but when mentioned, focused on blood tests and common side effects, including hypersalivation and weight gain.

**Conclusions:**

This is the first systematic review exploring patients’ subjective experiences of clozapine for TRS. Findings suggest that patients generally have a favorable experience when being treated with clozapine. However, conclusions are limited by the risk of bias, particularly survivorship bias. High-quality longitudinal studies exploring patients’ experiences of clozapine are indicated for the future.

## Introduction

Schizophrenia affects approximately 24 million people worldwide, with a prevalence of 0.45% in adults,^1^ and is associated with a reduced life expectancy of 15 years compared with the general population.^2^ Approximately 20% to 30% of schizophrenia patients meet the definition of treatment resistance,^3,4^ broadly defined as an inadequate response to 2 antipsychotics each at an adequate dose and duration.^5^ Treatment resistance is particularly associated with poor prognosis, including deteriorated social functioning^6^ and an increased risk of suicide.^7^

Clozapine is the only licensed treatment for those with treatment-resistant schizophrenia (TRS) and the only medication with strong evidence for efficacy in TRS.^8,9^ However, it is under-utilized^10,11^ with an average delay of 5 years from the time patients first meet the criteria for TRS to when clozapine is initiated.^12^ There are several perceived barriers to the use of clozapine that likely contribute to these delays as well as lower rates of prescribing.^10,13^ For example, side effects, such as myocarditis,^14^ diabetes,^15^ and blood dyscrasias^16^ have been reported as barriers for clinicians to prescribe clozapine. Other clinician barriers include mandatory blood testing^17^ as well as a general lack of training and experience.^18,19^

In contrast, to date, there has been less emphasis on patients’ experiences of clozapine.^20^ Where patients have been directly asked, there is some evidence to suggest that they report more positive experiences of clozapine^21,22^ and fewer concerns than clinicians would expect.^23^ Through a systematic review, we sought to determine the attitudes and experiences of patients with TRS with direct experience of clozapine.

## Methods

The systematic review was conducted according to the Preferred Reporting Items for Systematic Reviews and Meta-Analyses (PRISMA) 2020 guidelines^24^ and the protocol was preregistered on PROSPERO (CRD42021298019).^25^ To identify relevant studies, searches were conducted up to November 2021 on the electronic databases of Embase, Medline, PsychInfo, and PubMed. The following search terms were used: schizophrenia OR schizoaffective OR schizophreniform OR psychosis OR schizophrenia spectrum disorder AND clozapine AND patient* OR service user* OR consumer* OR client* OR user* AND attitude* OR use OR barrier* OR finding* OR experience* OR view* OR opinion* OR perception* OR tolerance (see Supplementary Table S1 for the full search strategies). The reference lists of related reviews were also examined for any relevant studies.^17,21^

### Eligibility Criteria

Inclusion criteria were (1) original research published in a peer-reviewed journal, (2) adult populations (18 years or older) with a diagnosis of TRS including schizophrenia, schizoaffective disorder, schizophreniform disorder, and related psychotic disorders, (3) patients with direct experience of clozapine treatment, and (4) English language studies. Exclusion criteria were (1) indirect reports of the patient experience of clozapine, for example, from clinicians or family members, (2) reviews and meta-analyses, and (3) conference papers, lecture notes, case series, or editorials. Titles and abstracts were initially screened, and the full texts of the remaining studies were assessed for eligibility. The search window was 1956 (the year that clozapine was synthesised^26^) to 2021. Searches were conducted independently by 2 researchers (S.P. and B.M.) and any discrepancies were resolved by a third rater (G.B.).

### Data Extraction

The following variables were extracted (1) publication details; (2) study design; (3) demographics; (4) diagnoses and assessment; (5) sample size, measures and analyses used, response rate; and (5) clozapine treatment. Data were extracted by a researcher (S.P.) and independently verified by another (B.M.).

### Data Synthesis

Findings were grouped into the following themes: positive experiences, negative experiences, and side effects. Due to anticipated methodological heterogeneity, results were narratively summarized and visually represented using heat maps. For each theme, we focused on the top 10 most frequently reported items.

### Quality Assessment

Quality assessment was conducted using an adapted version of the Newcastle-Ottawa Quality Assessment Scale for cross-sectional studies^27^ (see Supplementary Table S2). The scale measures study quality including the representativeness of the sample, sample size, nonrespondents, and the assessment of the outcome. One item “the ascertainment of the exposure (risk factor)” was removed as it did not relate to the included studies and one item was added “selection bias (clozapine use)” to assess survivorship bias. The adapted tool contains 7 items and is scored out of a maximum of 9. Studies were categorized as high (6–9), medium (3–5), and low quality (0–2). Studies were rated by the primary researcher (S.P.) and verified by the second rater (G.B.).

## Results

### Search Results

A total of 1040 records were included through database searching and after duplicates were removed, 916 records were screened for eligibility from their title and abstract. Six relevant studies^8,23,28–31^ were identified by searching the reference lists of the included studies and another related review.^17^ Thirty-one studies were reviewed in full. Thirteen studies met eligibility and were included^23,30,32–42^ (see Supplementary figure S1, PRISMA flow diagram).

### Study Characteristics

Table 1 summarizes the study and participant characteristics. Of the 13 studies, the year of publication was between 1996 and 2021. Sample sizes ranged between 10 and 570 and the pooled sample size of patients treated with clozapine was 1487. Of the total sample, 999 (67%) were male. Six studies were conducted on outpatients,^23,32,34,35,37,38^ 2 on inpatients,^30,40^ 4 a mix of both,^36,39,41,42^ and 1 did not report this.^33^ Four studies reported the participants’ ethnicity.^23,30,36,42^ In 3 studies, the most common ethnicity was white (75% to 90%),^23,30,42^ and in 1 study it was black (71%).^36^ Response rates, where reported, were 29% to 94%. The participants’ mean age ranged between 33 and 55 years old. The mean duration of clozapine use was 3 to 152 months, and the mean daily dose was 196 to 540 mg. Seven studies recruited patients that had taken clozapine for a minimum amount of time: 6 months,^41,42^ 3 months,^30,40^ or 1 month.^34,37,39^ Three studies were recruited from clozapine clinics.^23,36,38^ Where reported, all participants were diagnosed with schizophrenia or schizoaffective disorder. Nine studies used a quantitative design,^23,30,34,36–41^ 1 a qualitative design,^32^ and 3 a mixed design.^33,35,42^

**Table 1.** Study and Participant Characteristics

Authors (Year)	Country	Sample Size	Response Rate % (*n*)	Quality Assessment	Participants		
					Age *M* (range)	Males	Females
Quantitative studies							
Kim et al. (2006)^34^	South Korea	57	—	Medium	34	36	21
Qurashi et al. (2015)^30^	UK	56	84 (56 of 67)	Low	38 (22–59)	56	0
Sharma et al. (2021)^36^	US	86	92 (194 of 211)	Medium	42 (20–72)	56	30
Siskind et al. (2017)^37^	Australia	257	—	Medium	37	189	68
Sloan et al. (1997)^38^	Ireland	28	—	Low	33 (19–65)	19	9
Takeuchi et al. (2016)^39^	Japan	100	94 (100 of 106)	Medium	45	53	47
Taylor et al. (2000)^23^	UK	570	44 (570 of 1284)	Low	—	361	209
Verma et al. (2021)^40^	India	52	–	Low	34	26	26
Waserman and Criollo (2000)^41^	Canada	130	77 (130 of 168)	Low	39 (19–67)	99	31
Qualitative and mixed method studies							
Angermeyer et al. (2001)^32,^^a^	Germany	80	77 (80 of 104)	Low	—	48	32
Hodge and Jespersen (2008)^33,^^b^	Australia	27	35 (27 of 77)	Low	35 (23–49)	18	9
Murphy et al. (2018)^35,^^b^	Australia	10	29 (10 of 35)	Low	55 (39–70)	7	3
Wolfson and Paton (1996)^42,^^b^	UK	34	85 (34 of 40)	Low	36 (21–60)	31 ^c^	9^c^

^a^Qualitative design.

^b^Mixed methods design.

^c^Based on total sample.

### Quality Assessment

Four studies were rated as medium quality and the remaining nine studies as low quality. Sample sizes were only justified in 1 study. In all studies, there was no comparability between respondent groups and self-reporting was employed. In addition, sample sizes were generally small, there was no blinding of interviewers and control groups were limited. Where reported, studies only included clozapine patients that were taking clozapine at the time of recruitment (see Supplementary Table S3).

### Positive Experiences of Clozapine

**Fig. 1.**
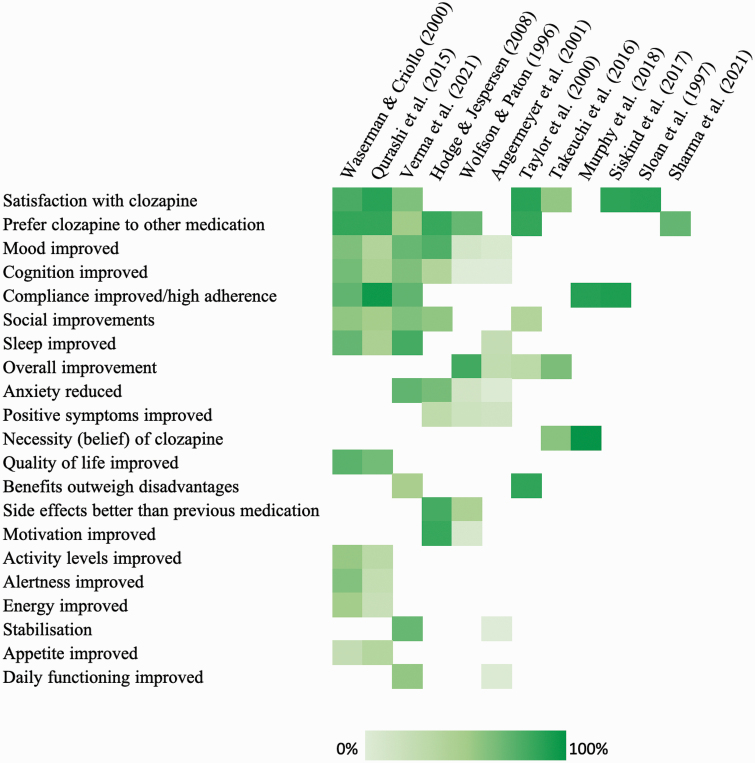
Positive experiences of clozapine. Items were included if reported by 2 or more studies. Each cell represents item percentages. Rows were ordered by the greatest number of items reported at the top. Columns were ordered by the greatest number of items reported per study to the left. Darker colors represent a higher proportion of patients endorsing the experience. Sloan et al.^38^ findings were based on the mean Client Satisfaction Questionnaire (CSQ-8) score, all other studies reported findings as percentages.

Positive experiences of clozapine were reported in 12 studies (figure 1). Across 7 studies, 58% to 90% of patients were satisfied with being on clozapine.^23,30,37–41^ Preference for clozapine, over other antipsychotic medications, was reported by 54% to 86% of patients.^23,30,33,36,40–42^ Specific improvements included improved mood (11% to 78%)^30,32,33,40–42^ and improved cognition (5% to 68%).^30,32,33,40–42^ Patients reported improved medication adherence in 5 studies (73% to 96%).^30,35,37,40,41^ Patients also reported improvements in ability to socialize (43% to 64%),^23,30,33,40,41^ sleep (28% to 81%),^30,32,40,41^ overall improvement (30% to 82%),^23,32,39,42^ anxiety (8% to 73%),^32,33,40,42^ and positive psychotic symptoms (19% to 33%).^32,33,42^

### Negative Experiences of Clozapine

**Fig. 2.**
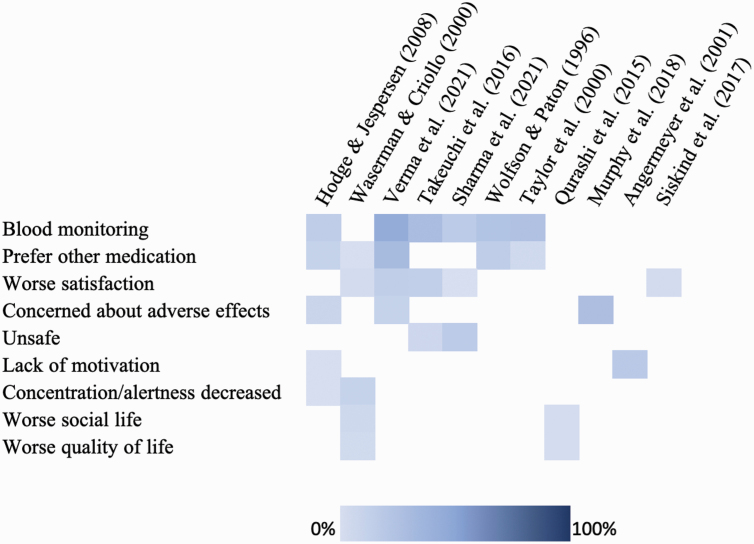
Negative experiences of clozapine. Items were included if reported in 2 or more studies. Each cell represents item percentages. Rows were ordered by the greatest number of items reported at the top. Columns were ordered by the greatest number of items reported per study to the left. Darker colors represent a higher proportion of patients endorsing the experience. A negative experience of blood monitoring was classified as being minded “*a little/medium amount*” by Hodge and Jespersen^33^ and “*somewhat/very much*” by Wolfson and Paton.^42^

Negative experiences of clozapine were reported in 11 studies (figure 2) which were mostly related to side effects, described below. Issues related to blood monitoring were reported by 18% to 46% of patients.^23,33,36,39,40,42^ In 5 studies, patients reported a preference for other medications over clozapine (3% to 33%).^23,33,40–42^ Five studies reported on patients’ dissatisfaction with clozapine (2% to 17%).^36,37,39–41^ Patients were concerned about adverse effects in 3 studies (11% to 30%)^33,35,40^ and felt that clozapine was unsafe in 2 studies (9% to 19%).^36,39^ Other negative experiences reported were a lack of motivation (3% to 21%),^32,33^ a decrease in concentration or alertness (3% to 14%),^33,41^ worse social life (4% to 8%),^30,41^ and worse quality of life (4% to 6%).^30,41^

### Side Effects

**Fig. 3.**
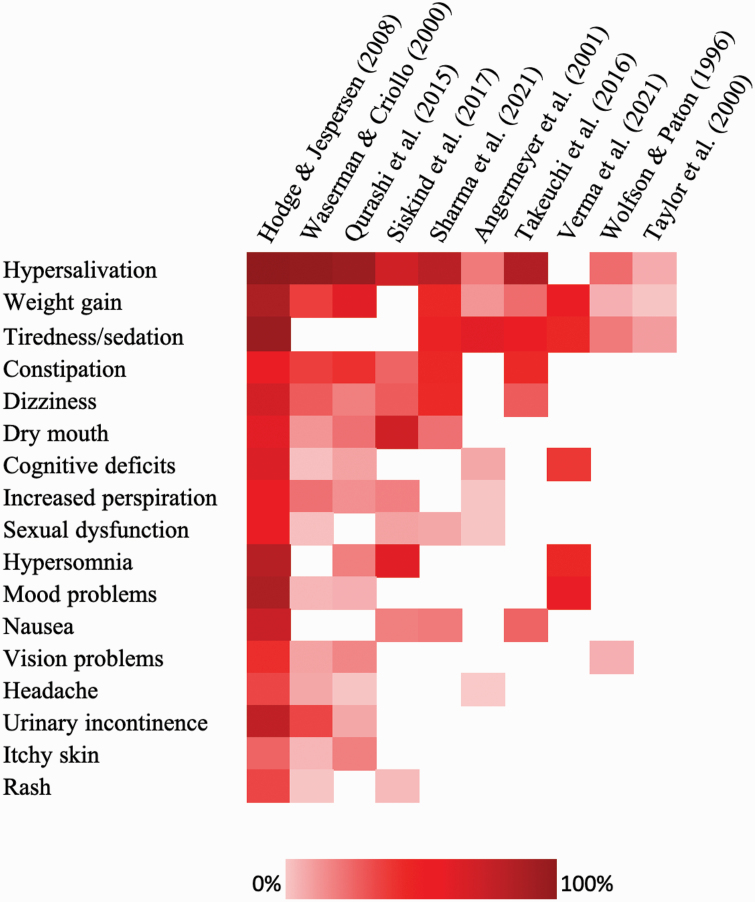
Side effects of clozapine. Items were included if reported in three or more studies. Each cell represents item percentages. Rows were ordered by the greatest number of items reported at the top. Columns were ordered by the greatest number of items reported per study to the left. Darker colors represent a higher proportion of patients endorsing the experience.

Side effects of clozapine were reported in 10 studies (figure 3). The most widely reported side effect was hypersalivation, reported by 10% to 89% of patients across 9 studies.^23,30,32,33,36,37,39,41,42^ Weight gain was reported in 5% to 78% of patients across 9 studies.^23,30,32,33,36,39-42^ Tiredness or sedation was reported by 13% to 85% of patients^23,32,33,36,39,40,42^ and constipation by 26% to 52%.^30,33,36,37,39,41^ Other reported side effects included dizziness (20% to 63%),^30,33,36,37,39,41^ dry mouth (15% to 64%),^30,33,36,37,41^ cognitive deficits (6% to 59%),^30,32,33,40,41^ increased perspiration (5% to 52%),^30,32,33,37,41^ sexual dysfunction (5% to 52%),^32,33,36,37,41^ and hypersomnia (20% to 74%).^30,33,37,40^

### Comparison Studies

Three studies compared clozapine patients with those taking other antipsychotics.^34,36,37^ One study compared clozapine with risperidone and found no significant differences between the groups, in terms of positive subjective response and attitude toward the medications.^34^ In another study, clozapine was compared with ten other antipsychotics.^36^ Clozapine patients reported more side effects, but the groups did not differ in terms of satisfaction, quality of life, and social ability.^36^ Finally, data from the 2010 Australian Survey of High Impact Psychosis was used to compare those taking clozapine with other antipsychotics.^37^ Satisfaction was highest for clozapine compared to zuclopenthixol decanoate (long-acting injectable) and olanzapine but not quetiapine. Adherence was highest for clozapine except compared with zuclopenthixol decanoate.^37^

## Discussion

We undertook a systematic review of patients’ attitudes and experiences of clozapine for TRS. Based on a narrative synthesis of 13 studies, our main findings were that most patients reported positive experiences of clozapine including overall satisfaction and specific improvements in mood, cognition, and anxiety. Negative experiences were less common and when reported, focused on dissatisfaction with clozapine for some patients, issues related to blood monitoring and side effects. Common side effects included hypersalivation, weight gain, sedation, constipation, and dizziness.

Satisfaction with clozapine was reported in 7 studies.^23,30,37–41^ Patients reported that they preferred to stay on clozapine,^23^ had greater satisfaction with clozapine compared with previous medications^30^ and preferred clozapine over other medications.^23,33,40–42^ Further, patients believed that the benefits of clozapine outweighed the disadvantages.^23,40^ Overall, there appears to be general acceptability for clozapine, but further research is needed to understand the reasons behind this such as comparing clozapine patients with discontinuers.

### Comparison With Previous Research

There has been limited research exploring patients’ satisfaction with clozapine and most studies have included the views of clinicians instead.^20^ In a systematic review and meta-analysis of 30 randomized controlled trials (RCTs) on the effectiveness of clozapine in schizophrenia, patients treated with clozapine exhibited greater clinical improvement and were more satisfied compared with other antipsychotics.^22^ However, the review included only 3 trials that reported on patients’ satisfaction and future trials are encouraged to measure patients’ subjective experiences of clozapine. A recent review examined the educational needs of clozapine users and their relatives across 30 studies,^21^ including seven from the current review.^23,32,33,35,39,41,42^ The results were broadly in keeping with our own, with most patients preferring clozapine to previously prescribed antipsychotics and reporting benefits in sleep, anxiety, and mood.

Patient and clinician views of clozapine treatment tend to differ,^20^ with clinicians underestimating patient satisfaction towards clozapine.^33^ Seventy-one percent of clinical staff (*n* = 144) surveyed within an inner London NHS trust indicated that patients were *more* satisfied with clozapine than with other atypical antipsychotics.^43^ Conversely, 66% of psychiatrists (*n* = 100) in Denmark believed that patients taking clozapine were *less* satisfied with their treatment compared to those taking other atypical antipsychotics.^44^ This inconsistency may have been due to one study having a higher proportion of clinicians (mostly trainee psychiatrists) that had never initiated clozapine^43^ compared with the other.^44^ However, a recent US study of 143 psychiatrists with an average of 20 years post-residency rated patients’ satisfaction with clozapine as over 70% compared with other antipsychotics.^45^ There is a suggestion that patients themselves are the biggest barrier to clozapine adoption, with concerns regarding blood tests and side effects.^43,46^ However, in the current review patients reported overall positive experiences on clozapine and in some cases, preferred it to other medications.

In the current review, 6 studies reported patients’ improvements in mood.^30,32,33,40–42^ In a systematic review of 25 RCTs, clozapine was superior to other antipsychotics in reducing positive symptoms, however, it only improved negative symptoms in the short term.^47^ The negative symptoms of schizophrenia are often the most difficult symptoms to treat and assessing them can be challenging.^48^

Regarding the negative experiences of clozapine in the current review, 5 studies^36,37,39–41^ reported that patients were dissatisfied with their clozapine treatment although to a lesser extent than those who were satisfied. In 6 studies,^23,33,36,39,40,42^ patients reported a dislike of blood tests that are required due to the risk of developing clozapine-induced agranulocytosis and neutropenia.^49^ In previous studies, clinicians considered blood monitoring to be one of the biggest issues for patients taking clozapine.^20,50^ A recent systematic review of 15 studies highlighted blood tests as a major barrier to the effective use of clozapine by clinicians.^17^ However, whilst some patients in the current review were troubled by the need for blood monitoring, other patients were not and realized it was a necessary part of their treatment which was consistent across 4 studies.^23,33,40,42^ The findings highlight the concerns of clinicians regarding mandatory blood tests may be inflated.^43,44^ A recent study has demonstrated the potential impact of revising clozapine monitoring requirements which may lessen the need for excessive blood monitoring and reduce discontinuation.^51^ Further research is required to establish the minimum monitoring requirements that balance patient safety and burden.

Hypersalivation and weight gain were the most widely reported side effects in the current review which is consistent with previous findings.^14,15^ Sedation was also common,^23,32,33,36,39,40,42^ which is consistent with previous research.^52,53^ In a cohort study of 316 TRS patients regarding the reasons for discontinuing clozapine, sedation was the most common patient-led decision.^54^ Clinicians should be aware of the side effects reported by patients on clozapine as they can be a major factor in discontinuation,^54^ especially as most can often be managed effectively through pharmacological and non-pharmacological interventions.^14^ For example, hypersalivation can often be improved by elevating the head at night and/or the use of anticholinergic medications and metoclopramide.^14^ Two studies in the current review reported that patients particularly appreciated the absence of extrapyramidal side effects^32,42^ which are known to be less common in clozapine.^55^ It was not possible to determine which other side effects or aspects of blood monitoring may have the biggest impact on the tolerability of clozapine and future research is indicated to explore this.

### Strengths

This is the first systematic review to focus on the subjective patient experience of clozapine and the review followed the PRISMA guidelines.^24^ Furthermore, a quality assessment was completed for all studies by following standardized guidelines to help ascertain the risk of bias.^27^ Previous reviews on this topic have tended to compare the patient experience of clozapine with clinicians.^17^ By focusing on patients, the review allows for a greater depth of understanding of their experiences.

Subjective reports regarding treatment have been met with criticism by some due to concerns of reliability^56^ as schizophrenia patients may have distorted or impaired cognition.^42^ However, studies have shown that patients can reliably report their subjective experiences including their quality of life and well-being.^57^ Furthermore, diagnoses are often reached using patients’ subjective experiences of their symptoms, such as hallucinations, which are not treated with the same skepticism.^56^ Therefore, patients’ views, such as in the current review, are necessary to move toward a patient-centered approach to improve outcomes and medication adherence.^58^

### Limitations

The studies included in this review used several different measures, such as bespoke questionnaires, therefore, it was difficult to synthesize the study results and it was not possible to conduct a meta-analysis. The response rates were acceptable,^59^ except in 3 studies that had less than 50%.^23,33,35^ Further, there are limitations regarding the representativeness of the study samples. Only 4 studies reported ethnicity,^23,30,36,42^ with most patients being white, therefore, the findings may be at risk of underrepresenting the views of patients of other ethnicities.^60^

Most participants had been taking clozapine for at least one year and were taking clozapine at the time of recruitment. This limited our ability to explore patient-related factors that can contribute to the underuse of clozapine due to treatment discontinuation, such as monitoring or adverse effects.^17^ Furthermore, our results are likely to reflect survivorship bias in favor of patients who tolerated and responded well to clozapine.^61^ Future studies would benefit from including patients who have discontinued clozapine. Further, it would be valuable to capture the views of family members or caregivers to ascertain the degree of agreement. The sample sizes were generally small, with most studies having under 100 participants, random sampling and control groups were limited, and the studies were at medium to high risk of bias which limits the generalizability of the results.

### Implications for Clinical Practice

Clozapine is the most effective antipsychotic for those with TRS,^47^ yet evidence suggests it is under-utilized with an average delay of 3 to 5 years before it is offered to patients.^8,12^ A potential barrier to adoption is clinicians’ perception that clozapine is a difficult medication to manage and that patients will not tolerate blood monitoring or side effects.^17^ Our findings suggest that patients had broadly positive experiences with clozapine and that blood monitoring and side effects were generally well tolerated. It is important for clinicians to consider these patient experiences to avoid a potential bias toward not offering clozapine. During patient discussions, this bias could lead clinicians to inadvertently deter patients who could potentially benefit from its use.^47^

### Future Research

All the studies in the current review used self-report measures for eliciting patient views.^56^ However, they are susceptible to bias and multiple self-report measures on separate dimensions can be used instead.^62^ Furthermore, the use of validated standardized measures would aid interpretability, as well as synthesizing the literature.^56^ Larger sample sizes with random sampling, control groups, and follow-up periods are encouraged. Finally, existing studies have tended to underrepresent black and minority ethnic individuals; future studies should aim to recruit a more diverse sample to increase the generalizability of findings.

In conclusion, patients’ experiences of clozapine were mainly positive with high levels of satisfaction and preference for clozapine compared with other medications. Blood monitoring was mentioned as a negative experience by some patients, although the majority found it tolerable, alongside common side effects, such as hypersalivation, weight gain, and sedation. Accepting the limitations of this systematic review, particularly the risk of survivorship bias, the findings further our understanding of patients’ experiences of clozapine. Findings may help enhance patients’ experiences with clozapine using a shared decision-making approach to improve adherence and lead to better outcomes. Further studies are needed in this area with high-quality longitudinal designs, including comparisons with patients that have discontinued treatment.

## Supplementary Material

sgac042_suppl_Supplementary_Materials
