# Supplementary material for: Patients’ Experiences of Clozapine for Treatment-Resistant Schizophrenia: A Systematic Review
Source: Schizophr Bull Open. 2022 Jul 10;3(1):sgac042. doi: 10.1093/schizbullopen/sgac042 (PMC11205966; doi:10.1093/schizbullopen/sgac042)
Supplement: sgac042_suppl_Supplementary_Materials [file sgac042_suppl_Supplementary_Materials.docx]

**Patients’ Experiences of Clozapine for Treatment-Resistant Schizophrenia: A Systematic Review**

**Supplementary Materials**

Steven Parkes^1^

Bethany Mantell^1,2^

Ebenezer Oloyede^1,2^*

Graham Blackman^1,2^*

1. Department of Psychosis Studies, Institute of Psychiatry, Psychology and Neuroscience, King’s College London, London, UK

2. South London and Maudsley NHS Foundation Trust, London, UK

* Joint senior authors

To whom correspondence should be addressed:

Steven Parkes, Institute of Psychiatry, Psychology and Neuroscience (IoPPN), King’s College London, 16 De Crespigny Park, London, SE5 8AF, UK; tel: 0207 848 0002; e-mail: [steven.j.parkes@kcl.ac.uk](mailto:steven.j.parkes@kcl.ac.uk)

**Table S1**. Search strategies for Embase, Medline, PsychInfo and PubMed databases.

| **Embase** | |
| --- | --- |
| 1 | Attitude/ OR incidental findings/ OR barrier*.mp. OR experience*.mp. OR view*.mp. OR opinion*.mp. OR perception*.mp. |
| 2 | clozapine.mp. OR Clozapine/ |
| 3 | patient/ OR consumer*.mp. OR "service user*".mp. OR client*.mp. OR user*.mp. |
| 4 | exp schizophrenia spectrum disorder/ OR schizophrenia*.mp. OR exp psychosis/ |
| 5 | 1 AND 2 AND 3 AND 4 |
| 6 | limit 5 to english language |
| **Medline** | |
| 1 | Attitude/ OR incidental findings/ OR barrier*.mp. OR experience*.mp. OR view*.mp. OR opinion*.mp. OR perception*.mp. |
| 2 | clozapine.mp. OR Clozapine/ |
| 3 | exp "schizophrenia spectrum and other psychotic disorders"/ OR schizophrenia*.mp. OR schizoaffective*.mp. OR schizophreniform*.mp. OR psychosis.mp. OR psychotic*.mp. OR schizophrenic*.mp. |
| 4 | exp Patients/ OR patient*.mp. OR consumer*.mp. OR "service user*".mp. OR client*.mp. OR user*.mp. |
| 5 | 1 AND 2 AND 3 AND 4 |
| 6 | limit 5 to (english language and yr="1956 -Current") |
| **PsychInfo** | |
| 1 | exp Attitudes/ OR barrier*.mp. OR experience*.mp. OR view*.mp. OR perception/ OR tolerance.mp. |
| 2 | clozapine.mp. OR Clozapine/ |
| 3 | patients/ OR consumer*.mp. OR "service user*".mp. OR exp Clients/ OR user*.mp. |
| 4 | exp Psychosis/ OR schizophrenia*.mp. OR psychosis.mp. OR psychotic*.mp. OR schizophrenic*.mp. |
| 5 | 1 AND 2 AND 3 AND 4 |
| 6 | limit 5 to (english language and yr="1956 -Current") |
| **PubMed** | |
| ("clozapine"[MeSH Terms] OR "clozapine"[All Fields] OR "clozapin"[All Fields] OR "clozapines"[All Fields]) AND ("Patients"[MeSH Terms] OR "service user*"[Text Word] OR "consumer*"[Text Word] OR "user*"[Text Word]) AND ("Attitude"[MeSH Terms] OR "use"[Text Word] OR "barrier*"[Text Word] OR "experience*"[Text Word] OR "view*"[Text Word] OR "perception*"[Text Word]) AND ("Schizophrenia Spectrum and Other Psychotic Disorders"[MeSH Terms] OR "psychosis"[Text Word] OR "schizophrenia*"[Text Word] OR "schizophreniform*"[Text Word] OR "schizoaffective*"[Text Word] OR "psychotic*"[Text Word] OR "schizophrenic*"[Text Word] OR "treatment-resistant schizophrenia"[All Fields] OR "treatment-resistant psychosis"[All Fields]) | |

**Table S2**. Adapted version of the Newcastle-Ottawa Quality Assessment Scale for cross-sectional studies.

| Selection (maximum 3 stars) | 1) Representativeness of the sample:   1. Truly representative of the average in the target population. * (all subjects or random sampling) 2. Somewhat representative of the average in the target population. * (non-random sampling) 3. Selected group of users. 4. No description of the sampling strategy.   2) Sample size:   1. Justified and satisfactory. * 2. Not justified.   3) Non-respondents:   1. Comparability between respondents and non-respondents characteristics is established, and the response rate is satisfactory. * 2. The response rate is unsatisfactory, or the comparability between respondents and non-respondents is unsatisfactory. 3. No description of the response rate or the characteristics of the responders and the non-responders. |
| --- | --- |
| Comparability (maximum 2 stars) | 1) The subjects in different outcome groups are comparable, based on the study design or analysis. Confounding factors are controlled.   1. The study controls for the most important factor (select one). * 2. The study control for any additional factor. * |
| Outcome (maximum 3 stars) | 1) Assessment of the outcome:   1. Independent blind assessment. ** 2. Record linkage. ** 3. Self report. * 4. No description.   2) Statistical test:   1. The statistical test used to analyze the data is clearly described and appropriate, and the measurement of the association is presented, including confidence intervals and the probability level (p value). * 2. The statistical test is not appropriate, not described or incomplete. |
| Selection bias (maximum 1 star) | 1. Clozapine use:    1. All of those with experience of clozapine were included e.g., currently prescribed, and previously prescribed/discontinued. *    2. Only those taking clozapine at the time of recruitment were included.    3. Not reported. |

The adapted version of the Newcastle-Ottawa Quality Assessment Scale^1^ is the same version used by Herzog et al.^2^ with one item removed: the ascertainment of the exposure (risk factor) and one item added: selection bias (clozapine use).

**Table S3**. Quality assessment of studies using an adapted version of the Newcastle-Ottawa Quality Assessment Scale (Table S2).

| **Authors (Year)** | **Representative Sample (1)** | **Sample Size (1)** | **Non-respondents (1)** | **Comparability (2)** | **Assessment Of Outcome (2)** | **Statistical Test (1)** | **Selection bias (1)** | **Total (9)** | **Quality Assessment** |
| --- | --- | --- | --- | --- | --- | --- | --- | --- | --- |
| Angermeyer et al. (2001) | (b) * | (b) | (b) | - | (c) * | (b) | (b) | 2 | Low |
| Hodge & Jespersen (2008) | (b) * | (b) | (b) | - | (c) * | (b) | (b) | 2 | Low |
| Kim et al. (2006) | (b) * | (b) | (b) | (a) * | (c) * | (a) * | (b) | 4 | Medium |
| Murphy et al. (2018) | (c) | (a) * | (b) | - | (c) * | (b) | (c) | 2 | Low |
| Qurashi et al. (2015) | (b) * | (b) | (b) | - | (c) * | (b) | (b) | 2 | Low |
| Sharma et al. (2021) | (b) * | (b) | (b) | (a) * | (c) * | (b) | (b) | 3 | Medium |
| Siskind et al. (2017) | (a) * | (b) | (c) | (a) (b) ** | (c) * | (a) * | (b) | 5 | Medium |
| Sloan et al. (1997) | (c) | (b) | (c) | - | (c) * | (b) | (b) | 1 | Low |
| Takeuchi et al. (2016) | (b) * | (b) | (c) | - | (c) * | (a) * | (b) | 3 | Medium |
| Taylor et al. (2000) | (b) * | (b) | (b) | - | (c) * | (b) | (b) | 2 | Low |
| Verma et al. (2021) | (b) * | (b) | (c) | - | (c) * | (b) | (b) | 2 | Low |
| Waserman & Criollo (2000) | (b) * | (b) | (b) | - | (c) * | (b) | (b) | 2 | Low |
| Wolfson & Paton (1996) | (b) * | (b) | (b) | - | (c) * | (b) | (b) | 2 | Low |

**Figure S1**. PRISMA 2020 flow diagram for systematic reviews.^3^

**References**

1. Wells GA, Shea B, O’Connell D, et al. The Newcastle-Ottawa Scale (NOS) for assessing the quality of nonrandomised studies in meta-analyses. Ottawa Hospital Research Institute. <http://ohri.ca/programs/clinical_epidemiology/oxford.asp>. Accessed 13 March, 2022.

2. Herzog R, Álvarez-Pasquin MJ, Díaz C, Del Barrio JL, Estrada JM, Gil Á. Are healthcare workers’ intentions to vaccinate related to their knowledge, beliefs and attitudes? A systematic review. *BMC Public Health.* 2013;13(1):154. doi:10.1186/1471-2458-13-154

3. Page MJ, McKenzie JE, Bossuyt PM, et al. The PRISMA 2020 statement: An updated guideline for reporting systematic reviews. *BMJ.* 2021;372:n71. doi:10.1136/bmj.n71
